# IL-12 ameliorates diabetic retinal neurodegeneration by activating microglial phagocytosis via the TREM2/DAP12 pathway

**DOI:** 10.1007/s12035-025-05512-1

**Published:** 2025-11-29

**Authors:** Wanyi Fang, Shanshan Liu, Yifei Wu, Ting Chen, Xiaohe Lu, Hui Chen

**Affiliations:** Department of Ophthalmology, Zhujiang Hospital, Southern Medical University, 253 Gongye Middle Avenue, Guangzhou, 510280 GuangDong China

**Keywords:** Retina, Interleukin-12, Microglia, TREM2, DAP12

## Abstract

Diabetic retinopathy (DR) is a common neurovascular complication of diabetes and a leading cause of vision loss in the advanced stages. Identifying therapeutic targets to prevent early progression of DR is critical for preserving visual function. Interleukin-12 (IL-12) has emerged as a potential therapeutic agent for early-stage diabetic retinal neurodegeneration. In this study, diabetic mouse models were established, followed by intravitreal injection of IL-12 to evaluate its effects using hematoxylin and eosin staining and RNA sequencing. IL-12 treatment partially prevented the thinning of the nerve fiber layer, ganglion cell layer, and total retina. Bioinformatics analysis of RNA sequencing data revealed enrichment of microglial signatures and enhanced phagocytic function. Western blotting analysis showed that IL-12 promoted the phosphorylation of signal transducer and activator of transcription 4 (STAT4) in microglia. Bioinformatics and quantitative reverse transcription polymerase chain reaction analyses demonstrated that STAT4 activation upregulated the transcription of phagocytosis-related genes, including triggering receptor expressed on myeloid cells 2 (TREM2) and DNAX-activating protein of 12 kDa (DAP12). In vitro and in vivo experiments confirmed that IL-12 upregulates the TREM2/DAP12 signaling pathway on the microglial membrane, enhancing microglial proliferation and phagocytic activity under high-glucose conditions. These findings indicated that IL-12 mitigates early neural injury in DR by promoting microglial phagocytosis through the upregulation of TREM2/DAP12.

**Supplementary Information:**

The online version contains supplementary material available at 10.1007/s12035-025-05512-1.

## Introduction

Diabetic retinopathy (DR), is one of the most serious ocular complications of diabetes and a leading cause of vision loss in adults [1, 2]. Current clinical treatments for DR, including retinal laser photocoagulation, vitrectomy, and medication, are limited in effectiveness, because most patients are diagnosed in the middle-to-late stages when retinal neural damage is often irreversible [3]. Therefore, understanding the mechanisms underlying early DR is crucial for delaying or preventing disease progression. Recent studies have shown that diabetic retinal neurodegeneration (DRN) precedes vascular degeneration and plays a central role in the early development of DR [4, 5]. Retinal ganglion cells (RGCs) are the primary pathological cells in optic neuropathy, and their structural and functional integrity plays a significant role in the progression of DRN [6–8]. Therefore, elucidating the mechanisms of RGC damage is essential.

Timely clearance of damaged or dead RGCs is essential for maintaining a healthy neural network in the central nervous system and depends on microglial phagocytosis [9, 10]. Microglia, which are the resident innate immune cells of the retina, monitor and regulate the retinal microenvironment through dynamic branching and stretching. They also mediate synaptic pruning and support synaptic development and plasticity. Microglia play crucial roles in immune responses, retinal nerve tissue development, and maintaining internal homeostasis [11–14]. Studies have shown that microglia can phagocytose RGC presynaptic terminals and that inhibiting CR3/C3-dependent microglial phagocytosis leads to synaptic remodeling defects [15]. Furthermore, microglia eliminate neurons via complement-mediated engulfment of non-apoptotic cells [16]. These findings indicate that retinal microglia efficiently and precisely clear dead cells through efferocytosis, thereby preventing inflammation caused by the release of toxic components or intracellular antigens and preserving normal tissue function [17]. The RGCs undergo degeneration and death in early DR, releasing their cellular contents into the surrounding environment. This activates inflammatory immune pathways, disrupts the blood-retinal barrier, and exacerbates retinal cell damage [8, 18, 19]. Therefore, identifying drugs that enhance microglial function is clinically important for inhibiting early DR progression and preserving visual function.

Interleukin-12 (IL-12), a heterodimeric cytokine produced by activated antigen-presenting cells such as dendritic cells and macrophages, consists of two protein subunits (p35 and p40) linked by disulfide bonds [20]. Owing to its strong anti-angiogenic and immune-activating properties, IL-12 has been widely used in clinical antitumor therapy [21]. Recently, its neuroprotective role has gained increasing attention. Recombinant human IL-12 p80 has been shown to enhance oligodendrocyte differentiation and promote axonal regeneration, facilitating functional recovery of damaged sciatic nerves [22]. In diabetic rats, intravitreal injection of nanoparticle-supported IL-12 significantly inhibited retinal neovascularization by downregulating vascular endothelial growth factor and matrix metalloprotein-9, while restoring the thickness of the ganglion cell layer (GCL) [23]. These findings suggest that IL-12 may be a potential therapeutic agent for early-stage DRN. However, its effects on the DRN in mice and the underlying mechanisms remain unclear.

The aim of this study was to investigate the effects and mechanisms of IL-12 treatment in early DR progression using in vivo diabetic mouse models and in vitro high-glucose microglial models, combined with transcriptome sequencing, to provide a scientific basis for identifying potential therapeutic targets for early-stage DR.

## Materials and Methods

### Diabetic Mouse Model and Intravitreal Injection

Specific pathogen-free C57BL/6 male mice, aged 6–7 weeks and weighing 20–24 g, were used in this study. Mice were housed at the Experimental Animal Center of Zhujiang Hospital, Southern Medical University, under controlled conditions (21 °C, 60% humidity, and 12 h light/dark cycle), with free access to food and water. All procedures were approved by the Animal Care and Use Committee of the Laboratory Animal Center at Zhujiang Hospital of Southern Medical University and were conducted per the Guide for the Care and Use of Laboratory Animals by the National Research Council.

In the modeling group, diabetes mellitus was induced by intraperitoneal injection of streptozotocin (STZ) at 50 mg/kg (dissolved in sodium citrate, 0.005 mL/g; Solarbio, Beijing, China) for 5 consecutive days [24], combined with a high-glucose, high-fat diet. The high-glucose and high-fat diet, comprising 66.5% standard mouse maintenance chow, 10% lard, 20% sucrose, 2.5% cholesterol, and 1% sodium cholate, was purchased from Xiaoshu YouTai Biotechnology Co., Ltd. (Beijing, China) with License No. SCXK (Beijing) 2018–0006. The control group received an equal volume of sodium citrate solution. A random blood glucose level above 16.7 mmol/L 2 weeks after injection indicated successful model establishment. Water intake, urine volume, and body weight were monitored and recorded in both groups.

Seven weeks after modeling, intravitreal injections were administered twice at 1-week intervals. The intravitreal injection volume was 1 µL. Mice were divided into three groups: a normal control group (CON; no injection), a diabetic group (DM; injected with phosphate-buffered saline [PBS]), and a diabetic + IL-12 group (DM + IL-12; injected with 100 ng/µL IL-12 [GLPBIO, Montclair, CA, USA] dissolved in PBS [Gibco, Grand Island, NY, USA]) [25]. To prevent infection, all mice received gatifloxacin eye drops (Laoboyuntang, Kunming, China) in the conjunctival sac 1 day before, 30 min before, and 1 day after the procedure. Anesthesia was induced by intraperitoneal injection of 0.015 mL/g tribromoethanol solution (Aladdin, Shanghai, China).

One week after the final injection, mice were euthanized under anesthesia with 3% pentobarbital sodium in strict accordance with animal care and use committee guidelines before sample collection.

### Histology and Immunostaining

Eyeballs were fixed in FAS eyeball fixation solution (Leagene, Beijing, China) for 24 h. Tissue dehydration, paraffin embedding, and sectioning were performed according to standard protocols [26]. For hematoxylin–eosin (HE) staining, tissue sections were incubated at 60 °C for 2 h and dewaxed three times with xylene, followed by hydration through a graded alcohol series. After hematoxylin staining for approximately 7 min, sections were differentiated in 1% acid alcohol, counterstained with eosin for 20 s, and dehydrated using gradient alcohol. The sections were then cleared with xylene and sealed with neutral resin. Images were captured using a 3DHistech bright-field scanner (3DHistech Ltd., Budapest, Hungary) and analyzed using Slide Viewer software. Retinal thickness was measured at multiple points and displayed in a spider plot. The optic disk was set as the ‘0’ reference; measurements were acquired superior (+) and inferior (−) at 400, 700, 1000, 1300, 1600, and 1900 μm from the optic disk.

Immunohistochemistry was performed on paraffin-embedded sections that were incubated, dewaxed, and rehydrated as described above. Sections were boiled in sodium citrate buffer (Boster, Wuhan, China) over medium heat for 20 min, then permeabilized with 0.3% Triton X-100 solution (Solarbio, Beijing, China) for 20 min. Endogenous peroxidase activity was blocked using a peroxidase blocker buffer (Zhongshan Jinqiao, Beijing, China) for 10 min, followed by blocking with 10% goat serum (Gibco, Grand Island, NY, USA) for 15 min. Sections were incubated with the primary antibody overnight at 4 °C, followed by incubation with biotinylated secondary antibodies diluted in 10% goat serum at room temperature for 10–15 min. Horseradish peroxidase-conjugated streptavidin (Zhongshan Jinqiao, Beijing, China) was applied for 10–15 min, and color development was performed using DAB Plus substrate (Zhongshan Jinqiao, Beijing, China).

For immunofluorescence staining, frozen sections and cell slides were incubated with antigen repair solution (Beyotime, Shanghai, China) for 10 min and permeabilized with 0.3% Triton X-100 solution for 20 min. Blocking was performed with 10% goat serum for 60 min, followed by overnight incubation with primary antibodies at 4 °C. Secondary antibodies were applied at room temperature for 1 h in the dark, followed by DAPI staining (Solarbio, Beijing, China). After sealing with nail polish, images were captured using a Nikon inverted fluorescence microscope (Nikon, Tokyo, Japan). The primary antigens used in this study are listed in Table 1.

**Table 1:** Antibodies for western blotting, immunohistochemistry and immunocytochemistry

Antibody	Dilution concentration	Supplier	Catalog no	protein molecular weight
Anti-IL-12RB1	IHC (1/200)ICC (1/200)	Abcam	ab256805	59kDa
Anti-Iba1 antibody	IHC (1/5000)ICC (1/200)	Abcam	ab283319	17kDa
Anti-TREM2	WB(1/1000)	Bioss	bs-2723R	33kDa
Anti-TYROBP	WB(1/1000)	Bioss	bs-12630R	12kDa
Anti-β-actin	WB(1/1000)	Fude	FD0060	42kDa
Anti-p-STAT4	WB(1/500)	Santa cruz	sc-136194	89kDa
Anti-STAT4	WB(1/1000)	CST	2653S	89kDa
Goat anti-Rabbit IgG (H + L) Cross-Adsorbed ReadyProbes Secondary Antibody, Alexa Fluor 488	IHC(1/300)ICC (1/300)	Invitrogen	R37116	-
Goat anti-Rabbit IgG (H + L) Cross-Adsorbed ReadyProbes Secondary Antibody, Alexa Fluor 594	IHC(1/300)ICC (1/300)	Invitrogen	R37117	-
Goat Anti-Mouse IgG (HL)—Alexa Fluor 555	IHC(1/300)ICC (1/300)	Abmart	M213408	-
Goat anti-Rabbit(HPR)	WB(1/8000)	Fude	FDR007	-
Goat Anti-Mouse(HPR)	WB(1/8000)	Fude	FDM007	-

Abbreviations: western blotting, WB; immunohistochemistry, IHC; immunocytochemistry, ICC.

### Bioinformatics Analysis

The retinas were placed in centrifuge tubes and frozen in liquid nitrogen. For total RNA extraction, 1 mL of Trizol was added to each tube, and the samples were ground thoroughly using a cryogrinder. The samples were shaken for 15 s after 200 µL of chloroform being added, left to stand for 10 min, and centrifuged at 12,000 rpm for 15 min at 4 °C. The aqueous phase was discarded, and 1 mL of 75% ethanol was added. Samples were centrifuged again at 12,000 rpm for 5 min at 4 °C. After discarding the supernatant, 20 µL of RNase-free water was added to dissolve the RNA, and purity and concentration were measured using an ultraviolet spectrophotometer.

Poly(A) RNA was enriched from total RNA using Oligo(dT), followed by random fragmentation, reverse transcription, DNA linker ligation, polymerase chain reaction (PCR) amplification, and purification. RNA quality was assessed using an Agilent 2200 TapeStation, and qualified samples were sent to Guangzhou Ruibo Biotechnology Co., Ltd. for sequencing. Retinal transcriptome sequencing was performed using an Illumina platform. Raw sequencing data were filtered, de-concatenated, and preprocessed to obtain clean data, which were aligned to the reference genome using HISAT2.

Differentially expressed genes (DEGs) were identified based on fold change (|log_2_(FoldChange)|> 1) and statistical significance(q-value < 0.05). Enrichment pathway analysis was conducted across all three groups (CON, DM, and DM + IL-12) to identify group-specific and shared pathways. Kyoto Encyclopedia of Genes and Genomes (KEGG) and Gene Ontology (GO) analyses were performed, with GO covering molecular functions, cellular components, and biological processes. Intersectional gene analysis between groups was conducted using the Venn online tool, and differential gene sets were further analyzed for cell composition and KEGG pathways using the Metascape platform.

To explore how signal transducer and activator of transcription 4 (STAT4), activated by IL-12, regulates the transcription of efferocytosis-related genes, we downloaded STAT4 chromatin immunoprecipitation sequencing (ChIP-seq) data from IL-12-stimulated natural killer (NK) cells (dataset GSE106137). After filtering and quality control, reads were mapped to the mouse reference genome with Bowtie2, peaks were called by MACS3, and differential peaks were identified using MAnorm2. STAT4-bound genes were then intersected with the up-regulated DEGs we previously obtained from mouse retinal RNA-seq, followed by a second intersection with 237 efferocytosis-associated genes curated from the GSEA database. This pipeline yielded a concise set of downstream efferocytosis candidates directly regulated by IL-12-activated STAT4.

### Cell Culture and Treatment

BV2 cells were donated by the Clinical Research Center of Zhujiang Hospital, Southern Medical University, and identified using ionized calcium-binding adapter molecule 1 (IBA-1) staining. Cells were cultured in a growth medium containing high-glucose Dulbecco's modified Eagle’s medium (DMEM), 10% fetal bovine serum (FBS), 1% penicillin–streptomycin solution, 1% nonessential amino acids, and 1% recombinant human fibronectin. Cultures were maintained at 37 °C in a humidified atmosphere of 5% CO_2_ and 95% air. Cells were passaged upon reaching 80% confluence. Before initiating the experiments, optimal glucose and IL-12 concentrations were determined. Following digestion, centrifugation, resuspension, and counting, the microglia were seeded into 24-well plates at a density of 2 × 10^5^ cells per well. Media were prepared with varying glucose concentrations (25, 45, 60, and 75 mM). The experimental groups included 45 mM glucose only, 45 mM + 1 ng/mL IL-12, 45 mM + 10 ng/mL IL-12, and 45 mM + 100 ng/mL IL-12. The medium was replaced every 36 h, and cells were harvested at 72 h.

### Quantitative Reverse Transcription Polymerase Chain Reaction(RT-qPCR)

Total RNA was extracted as previously described, and reverse transcription was performed using the AG Evo M-MLV reverse transcription premix kit. Next, qPCR was conducted using the SYBR ® Green Pro Taq HS premixed qPCR kit, with β-actin as the internal reference gene. A 20 µL reaction mixture was prepared on ice. Amplification and detection were carried out on an ABI fluorescence qPCR instrument using the following protocol: pre-denaturation at 95 °C for 30 s, followed by 40 cycles of denaturation at 95 °C for 5 s and annealing at 60 °C for 30 s. Primer sequences are listed in Table 2.

**Table 2:** The upstream and downstream primer sequences of the genes used in this study

Gene	Upstream Primers (5'—3')	Downstream Primers (5'—3')
TREM2	GCCTTCCTGAAGAAGCGGAA	TAGGCTAGAGGTGACCCACA
DAP12	CCTCCTGACTGTGGGAGGAT	TCACGGAAGAACAGTCGCAT
STAT4	GCACTCAGTAAGATGACGCAG	CCAGTAGGGTAAAGCAGTTCTG
IRF8	CGGGGCTGATCTGGGAAAAT	CACAGCGTAACCTCGTCTTC
CYBA	TGCCAGTGTGATCTATCTGCT	TCGGCTTCTTTCGGACCTCT
CORO1A	TCCTGATACCAACATTGTCTACC	TGAGACTCCTTGGAACTGAACA
TGM2	CGCAACAGGGCTTCATCTAC	CCCGACTACGGTTCTTCAGGA
ITGAM	GGGAAATGCCTTCAACAAACC	CTGTCTGCGTGCCCTCAAT
NCKAP1L	TGTCCGAAATAGCACGCAACA	ATCCCGAAATTCCATGACATCC
GAPDH	CAAAATGGTGAAGGTCGGTGT	GAGGTCAATGAAGGGGTCGTT
β-actin	CCCCTGAACCCTAAGGCCA	CGGACTCATCGTACTCCTGC

Primer specificity was verified using Primer-BLAST, and melting curve analysis confirmed the absence of primer dimers.

### Western Blotting (WB)

For protein extraction, a single retina was placed in a centrifuge tube with 80 µL of radio-immunoprecipitation assay (RIPA) lysis buffer containing 1% protease inhibitor, 1% phosphorylation inhibitor, and 1% phenylmethylsulfonyl fluoride, The tissue was homogenized using a cryogrinder then centrifuged at 14,000 rpm for 15 min at 4 °C to collect the supernatant. Protein concentration was measured using a bicinchoninic acid (BCA) assay kit. The protein solution was then mixed with loading buffer at a 4:1 ratio and heated at 100 ℃ for 10 min. For each sample, 20 µg of protein was subjected to sodium dodecyl sulfate–polyacrylamide gel electrophoresis (SDS-PAGE), and followed by transferred to polyvinylidene fluoride membranes using the wet transfer method. Membranes were blocked with 5% fetal bovine serum for 1 h and incubated overnight at 4 °C with primary antibodies (Table 1). After washing with Tris-buffered saline containing Tween 20, membranes were incubated with secondary antibodies for 1 h at room temperature. Protein detection was performed using an enhanced chemiluminescence and visualized using a chemiluminescence imaging system. Quantitative analysis was conducted using ImageJ software.

### Detection of Phagocytic Function

BV2 cells were cultured in 24-well plates at a density of 2 × 10^5^ cells per well. After 24 h, 1 µL/well of fluorescein isothiocyanate (FITC)-labeled fluorescent microspheres (Aladdin, Shanghai, China) were added, and cells were incubated in the dark for 6 h. Phagocytic function was assessed using flow cytometry and immunofluorescence staining. For flow cytometry, cells were digested, resuspended in PBS, and analyzed. A total of 10,000 cells per sample were recorded, grouped by side scatter (SSC), and analyzed via the FITC channel. For immunofluorescence staining, an anti-IBA-1 primary antibody (1:100) and a cyanine 3-conjugated secondary antibody (1:500) were used. FITC and cyanine 3 signals were visualized using a Nikon inverted fluorescence microscope.

### Statistical Analysis

All experiments were independently repeated at least three times. Data were analyzed and plotted using GraphPad Prism 9. We have conducted Shapiro–Wilk normality tests to assess sample distribution. For statistical comparison of two groups, student’s t-test were performed for normally distributed data, while Mann–Whitney tests were employed for non-normally distributed datasets. One-way analysis of variance (ANOVA) was performed to compare data among multiple groups using Tukey’s multiple comparison tests. Outliers were excluded by the robust regression and outlier removal (ROUT) method with Q = 5%. Sample sizes were justified by power analysis (α = 0.05, β = 0.2). Statistical significance was set at *p* < 0.05.

## Results

### Intravitreal Injection of IL-12 Preserves Retinal Structural Integrity in Early DRN mice

**Fig. 1:**
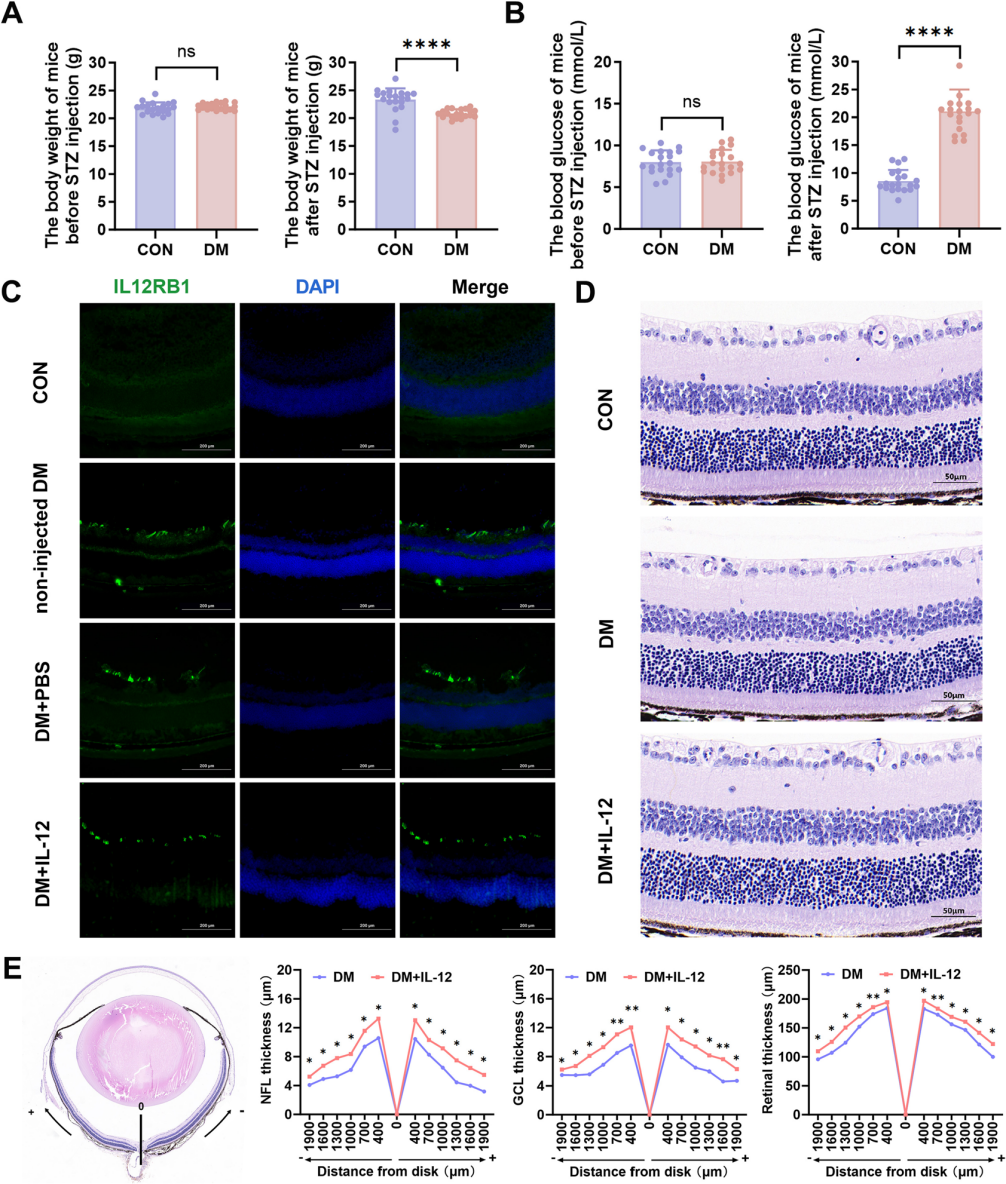
IL-12 ameliorates early diabetic retinal neurodegeneration in mice**.** (**A**) Body weight of mice before(*p* = 0.8932) and after(*p* < 0.0001) streptozotocin injection; n = 20. (**B**) Blood glucose levels of mice before(*p* = 0.3070) and after(*p* < 0.0001) streptozotocin injection; n = 20. (**C**) Expression and distribution of IL-12RB1 in the retinas of mice in the CON, DM, and DM + IL-12 groups; n = 3; scale bar = 200 μm. (**D**) Hematoxylin and eosin (HE) staining of retinal paraffin sections from the CON, DM, and DM + IL-12 groups; n = 3; scale bar = 50 μm. (**E**) Measurement and analysis of GCL, NFL, and total retinal thickness.The optic disk was set as the ‘0’ reference; measurements were acquired superior (+) and inferior (−) at 400, 700, 1000, 1300, 1600, and 1900 μm from the optic disk. 3 sections from different mice per group were accounted (n = 3 mice). (GCL: −1900: *p* = 0.0424, −1600: *p* = 0.0304, −1300: *p* = 0.0356, −1000: *p* = 0.0387, −700: *p* = 0.005, −400: *p* = 0.0011, 400: *p* = 0.0442, 700: *p* = 0.0401, 1000: *p* = 0.0131, 1300: *p* = 0.0111, 1600: *p* = 0.0016, 1900: *p* = 0.027; NFL: −1900: *p* = 0.0339, −1600: *p* = 0.0134, −1300: *p* = 0.0338, −1000: *p* = 0.0467, −700: *p* = 0.0433, −400: *p* = 0.0363, 400: *p* = 0.0288, 700: *p* = 0.0449, 1000: *p* = 0.0424, 1300: *p* = 0.0481, 1600: *p* = 0.0126, 1900: *p* = 0.0131; total retina: −1900: *p* = 0.0356, −1600: *p* = 0.0428, −1300: *p* = 0.0445, −1000: *p* = 0.047, −700: *p* = 0.0011, −400: *p* = 0.0445, 400: *p* = 0.0387, 700: *p* = 0.0023, 1000: *p* = 0.0421, 1300: *p* = 0.043, 1600: *p* = 0.0397, 1900: *p* = 0.0241) Unpaired t-tests were performed to compare data between two groups. ns, not significant, *p < 0.05, **p < 0.01, ****p < 0.0001. IL-12, interleukin-12; IL-12RB1, interleukin-12 receptor beta 1; CON, control group; DM, diabetic mice group; DM + IL-12, diabetic mice receiving an intravitreal injection of IL-12; GCL, ganglion cell layer; NFL, nerve fiber layer

Thinning of the retinal GCL, NFL, and the total retina is a well-established marker of early diabetic retinal pathological damage [27, 28]. These indicators were used to evaluate the effects of intravitreal IL-12 injection on the early DRN in mice. Prior to modeling, the mice were randomly assigned to normal (CON) and diabetic (DM) groups, with no significant differences in body weight (Fig. 1A) or blood glucose levels (Fig. 1B). Two weeks after modeling, the DM group showed significantly lower body weight(Fig. 1A, *p* < 0.0001) and higher blood glucose levels (Fig. 1B, *p* < 0.0001) compared with the CON group, confirming the successful induction of diabetes. Immunofluorescence staining (Fig. 1C) revealed minimal expression of Interleukin-12 receptor beta 1 (IL12RB1) in CON retinas. In contrast, IL-12RB1 expression was clearly observed in the DM group, regardless of IL-12 injection, and was predominantly localized in the GCL. Schematic diagram of mouse retinal layers was shown in Supplementary Fig. 1. HE staining showed restoration of the retinal layer thickness following IL-12 injection (Fig. 1D). To localize and quantify this effect, retinal thickness was measured at various distances from the optic nerve root. Diabetic mice showed significant thinning of the GCL, NFL, and total retina (Supplementary Fig. 2, all *p* < 0.05). However, the DM + IL-12 group exhibited significantly thicker GCL, NFL, and total retina compared with the DM group (Fig. 1E, all *p* < 0.05). In addition, at the early stage of diabetic retinopathy, there is no significant increase in TNF-α or IL-1β levels compared to the CON group. This suggests that there is no overt inflammatory response at this time point (Supplementary Fig. 3, all *p* > 0.05). Furthermore, IL-12 treatment did not induce any significant changes in the levels of these cytokines, indicating that IL-12 does not provoke an inflammatory response in this context (Supplementary Fig. 3, all *p* > 0.05).

### Bioinformatics Analysis of RNA Sequencing Reveals Enrichment of Microglial and Phagocytic Functions

**Fig. 2:**
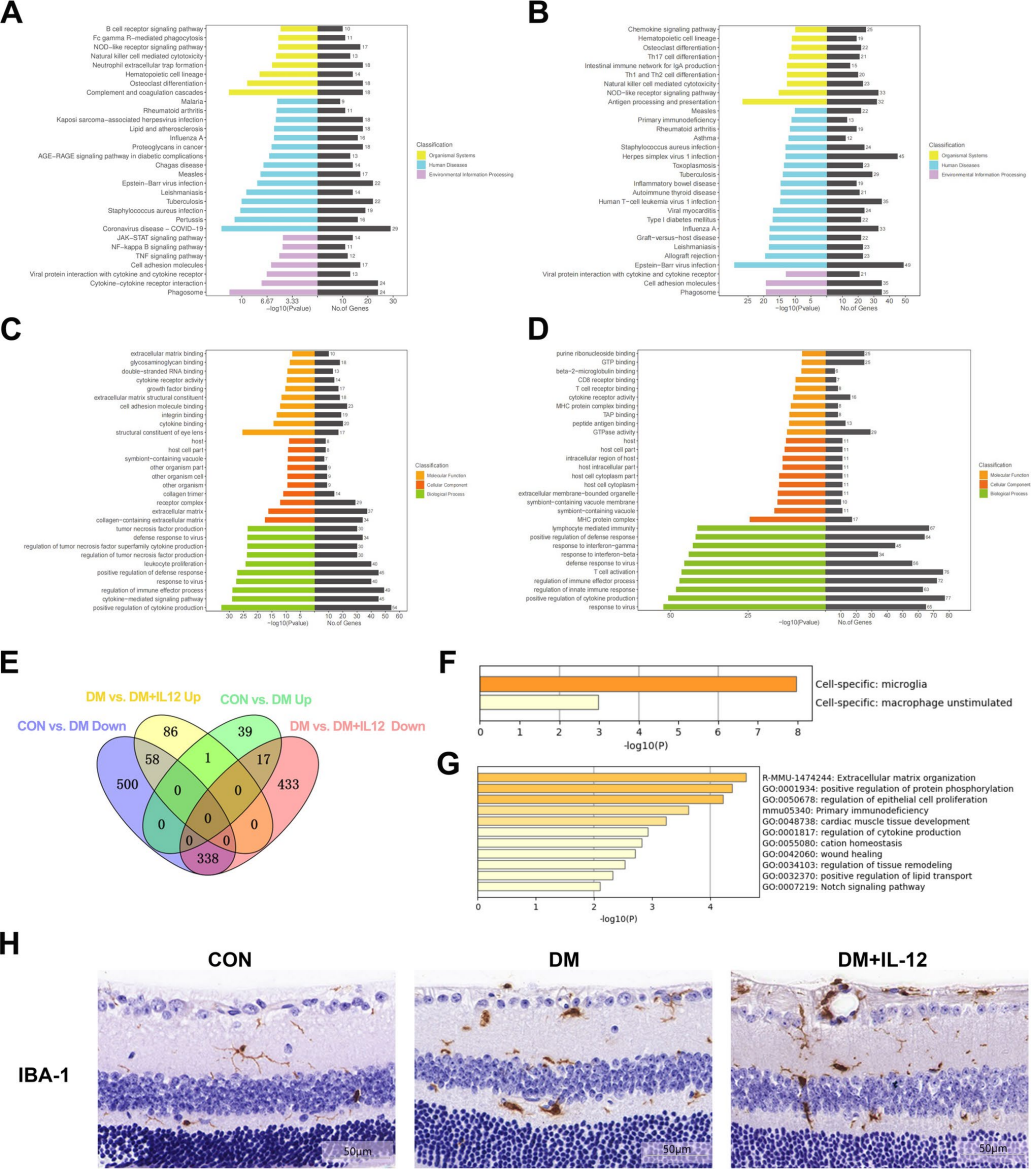
Bioinformatics analysis of RNA sequencing reveals enrichment in microglial and phagocytic pathways**.** (**A**) KEGG analysis of DEGs between the CON and DM groups; n = 3. (**B**) KEGG analysis of DEGs between the DM and DM + IL-12 groups; n = 3. (**C**) GO analysis of DEGs between the CON and DM groups; n = 3. (**D**) GO analysis of DEGs between the DM and DM + IL-12 groups; n = 3. (E) Venn diagram of DEGs across the three groups. (F) Cell type enrichment analysis of DEGs, and (**G**) pathway enrichment of DEGs, colored by *p*-values. (**H**) Immunohistochemical analysis of IBA-1 in the retinas of mice in the CON, DM, and DM + IL-12 groups; n = 3; scale bar = 50 μm. DEGs, differentially expressed genes; CON, control group; DM, diabetic mice group; DM + IL-12, diabetic mice receiving intravitreal injection of IL-12; GO, Gene Ontology; IBA-1, ionized calcium-binding adapter molecule 1; IL-12, interleukin-12; KEGG, Kyoto Encyclopedia of Genes and Genomes

Transcriptome sequencing revealed that, compared with the CON group, there were 366 upregulated genes and 3 downregulated genes in the DM group (Supplementary Fig. 4. A,C). In the DM + IL-12 group, 477 genes were upregulated and 25 were downregulated(Supplementary Fig. 4. B,D). KEGG and GO analyses were performed to assess the impact of DR status and IL-12 treatment on retinal biological pathways. Between the CON and DM groups, a total of 369 DEGs were identified. KEGG pathway enrichment analysis revealed 78 pathways significantly enriched (*p* < 0.05), with the most prominent including the complement and coagulation cascades, phagosomes, and cytokine-cytokine receptor interactions (Fig. 2A). In contrast, 502 DEGs between the DM and DM + IL-12 groups were enriched in 73 pathways (*p* < 0.05), with the most prominent of which were antigen processing and presentation, NOD-like receptor signaling, phagosomes, and cell adhesion molecules (Fig. 2B). Notably, the phagocytic pathway was the only top-five enriched pathway common to both comparisons (Figs. 2A, 2B).

Figures 2C and 2D show the results of GO analysis. Genes with |log_2_(FoldChange)|> 1 and *p* < 0.05 were selected as DEGs to capture a broad set of genes potentially, influenced by IL-12 during early DR. A Venn diagram comparing DEGs among groups (Fig. 2E) revealed that the DM group had 58 upregulated genes and 17 downregulated genes compared with the CON and DM + IL-12 groups, suggesting that these genes may be modulated by diabetes and partially reversed by IL-12 treatment. Enrichment analysis of these 75 DEGs showed predominant expression in microglia and unstimulated macrophages (Fig. 2F), with associated pathways primarily involving extracellular matrix organization and positive regulation of protein phosphorylation (Fig. 2G). Immunohistochemical analysis (Fig. 2H) further demonstrated that the retinas from normal mice contained few microglia, mostly in a branched, resting state, whereas the DM and DM + IL-12 groups showed increased microglial numbers with an amoeboid morphology, indicating activation. These findings suggest that DR status and IL-12 treatment may influence phagocytic function, likely through microglial activation.

### IL-12 Influences Microglial Phagocytic Function

**Fig. 3:**
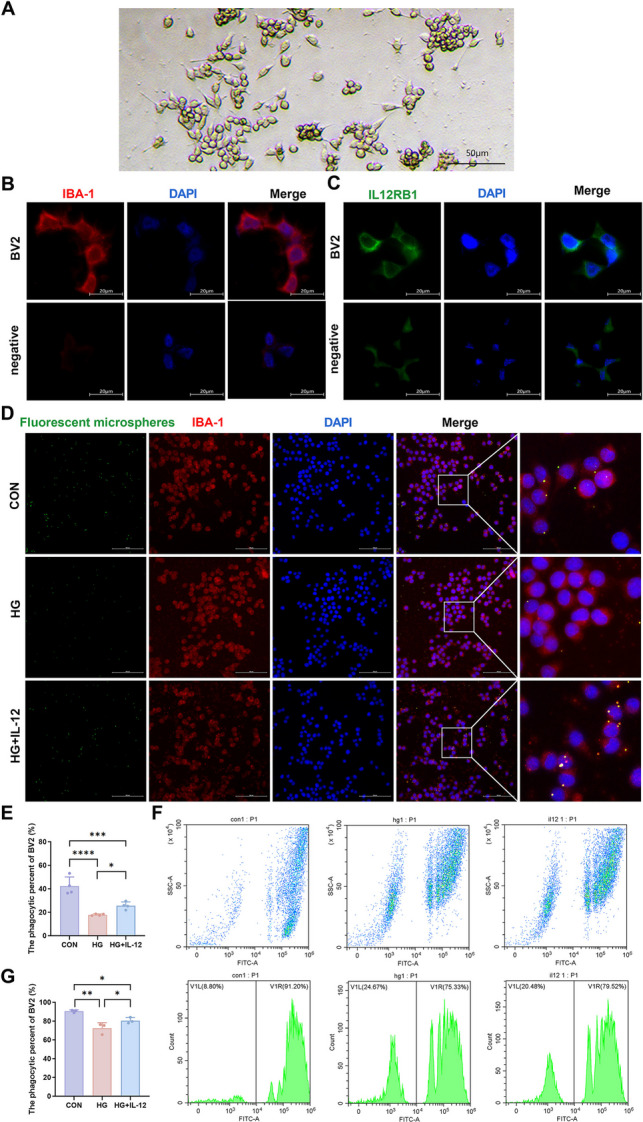
IL-12 enhances the phagocytic function of microglia in a high-glucose environment**.** (**A**) Microglial morphology observed under phase-contrast microscopy; n = 3; scale bar = 50 μm. (**B**, **C**) Immunofluorescence images showing IBA-1 and IL-12RB1 expression in microglia and negative controls; n = 3; scale bar = 20 μm. (**D**) Microglial phagocytosis of fluorescent microspheres visualized by immunofluorescence. Green, red, and blue represent microspheres, IBA-1, and nuclei, respectively; n = 4; scale bar = 100 μm. (**E**) Quantitative analysis of microglial phagocytosis based on immunofluorescence (CON vs. HG: *p* < 0.0001, CON vs. IL-12: *p* = 0.0009, HG vs. IL-12: *p* = 0.0496); n = 4. (**F**) Flow cytometric analysis of microglial phagocytosis. Cells to the left of the gate lacked microspheres, whereas those to the right contained at least one microsphere (10,000 cells analyzed); n = 3. (**G**) Quantitative analysis of microglial phagocytosis based on flow cytometry (CON vs. HG: *p* = 0.0014, CON vs. IL-12: *p* = 0.0211, HG vs. IL-12: *p* = 0.0466); n = 3. ANOVA tests were performed to compare data among CON, HG and IL-12 groups. ns, not significant; *p < 0.05, **p < 0.01, ***p < 0.001, ****p < 0.0001. IBA-1, ionized calcium-binding adapter molecule 1; IL-12, interleukin-12; IL-12RB1, interleukin-12 receptor beta 1

To determine whether microglial phagocytic function contributes to the IL-12-mediated reduction of RGC injury, we assessed the effects of IL-12 on microglial phagocytosis at the cellular level. Phase-contrast microscopy revealed that microglia exhibited irregular morphologies, including round and spindle-shaped forms (Fig. 3A), and were IBA-1-positive (Fig. 3B). Immunofluorescence staining confirmed the expression of IL-12 receptors on microglia (Fig. 3C). FITC-labeled monodisperse fluorescent microspheres were used to evaluate the phagocytic function. Immunofluorescence analysis showed that the average proportion of microglia co-localized with fluorescent microspheres was 42.32% in the CON group, 17.85% in the DM group, and 25.63% in the DM + IL-12 group (Fig. 3D). One-way ANOVA and multiple comparison analysis revealed statistically significant differences among groups (Fig. 3E). Flow cytometry further confirmed these findings: under normal conditions, 90.51 ± 1.43% of microglia engulfed fluorescent microspheres, whereas the phagocytic rate decreased to 72.40 ± 5.90% under high-glucose conditions (Fig. 3F, 3G). In the DM + IL-12 group, the rate increased to 80.48 ± 3.19%, significantly higher than that in DM group (Figs. 3F, 3G). These results indicate that high-glucose conditions suppress microglial phagocytic function, whereas IL-12 partially restores it. In addition, IL-12 enhanced microglial proliferation under high-glucose conditions but did not affect their migration (Supplementary Fig. 5).

### IL-12 Activates Microglial Phagocytic Function via the JAK-STAT-TREM2 Signaling Pathway

**Fig. 4:**
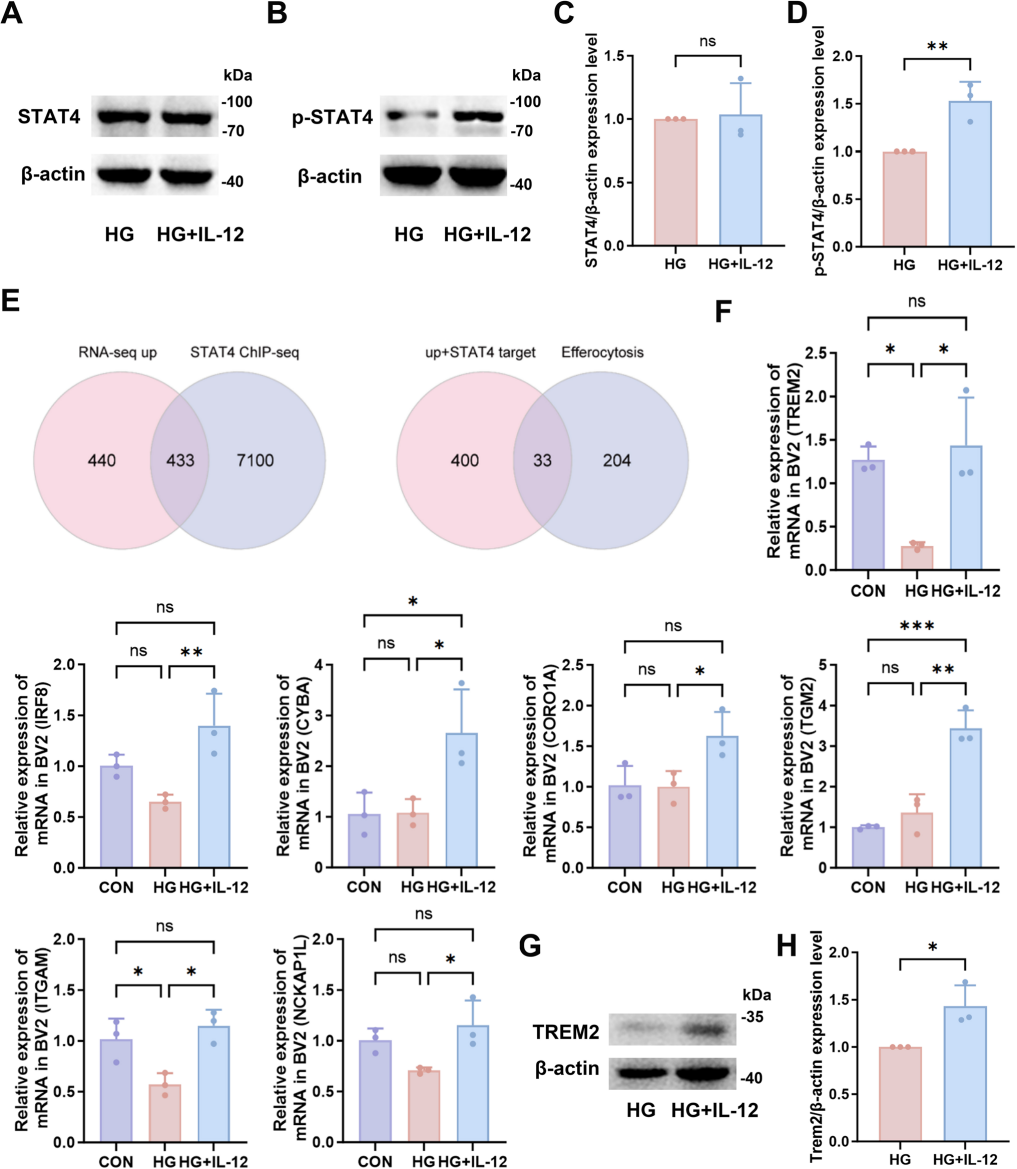
IL-12 activates microglial phagocytic function via the JAK-STAT-TREM2 signaling pathway**.** (**A**–**D**) WB analysis of STAT4 and p-STAT4 in microglia in the HG and HG + IL-12 groups(STAT4: *p* = 0.8188, p-STAT4: *p* = 0.0099); n = 3. (**E**) Venn diagrams showing overlap among upregulated RNA-seq data, STAT4 ChIP-seq data, and genes in phagocytic pathways; n = 3. (**F**) RT-qPCR analysis of candidate gene expression (TREM2: CON vs. HG: *p* = 0.0247, CON vs. HG + IL-12: *p* = 0.809, HG vs. HG + IL-12: *p* = 0.0122; IRF8: CON vs. HG: *p* = 0.1493, CON vs. HG + IL-12: *p* = 0.1091, HG vs. HG + IL-12: *p* = 0.0084; CYBA: CON vs. HG: *p* = 0.9984, CON vs. HG + IL-12: *p* = 0.0334, HG vs. HG + IL-12: *p* = 0.0356; CORO1A: CON vs. HG: *p* = 0.9959, CON vs. HG + IL-12: *p* = 0.0515, HG vs. HG + IL-12: *p* = 0.0463; TGM2: CON vs. HG: *p* = 0.5044, CON vs. HG + IL-12: *p* = 0.0005, HG vs. HG + IL-12: *p* = 0.0011; ITGAM: CON vs. HG: *p* = 0.0358, CON vs. HG + IL-12: *p* = 0.5975, HG vs. HG + IL-12: *p* = 0.0114; NCKAP1L: CON vs. HG: *p* = 0.1282, CON vs. HG + IL-12: *p* = 0.5211, HG vs. HG + IL-12: *p* = 0.0307); n = 3. (**G**, **H**) WB analysis of TREM2 expression in microglia in the HG and HG + IL-12 groups; n = 3, *p* = 0.0291. Unpaired t-tests were performed to compare data between HG and HG + IL-12 groups. ANOVA tests were performed to compare data among CON, HG and IL-12 groups. ns, not significant; *p < 0.05, **p < 0.01, ***p < 0.001. ChIP-seq, chromatin immunoprecipitation sequencing; HG, high-glucose group; HG + IL-12, microglia treated with high glucose and IL-12; STAT, signal transducer and activator of transcription; TREM2, triggering receptor expressed on myeloid cells 2

IL-12 exerts its cellular effects primarily through the classical STAT4 phosphorylation pathway. Upon binding to the IL-12 receptor on the cell membrane, IL-12 transmits signals to tyrosine, which phosphorylates and activates Janus kinase 2 (JAK2) and tyrosine kinase 2 (TYK2), leading to further STAT4 activation[29]. In this study, WB (Figs. 4A, 4B) showed no significant change in total STAT4 protein levels following IL-12 intervention (Fig. 4C, *p* > 0.05); however, phosphorylated STAT4 (p-STAT4) levels increased significantly (Fig. 4D, *p* < 0.01). To identify phagocytosis-related genes activated via IL-12-mediated STAT4 signaling, we mined STAT4 ChIP-seq data from IL-12-stimulated NK cells (GSE106137). After stringent filtering, 7,533 STAT4-bound genes were recovered. Intersection with the retinal RNA-seq up-regulated DEGs yielded 433 high-confidence candidates. To focus on efferocytosis, these were further filtered against 237 GSEA-defined efferocytosis genes, resulting in 33 direct downstream targets of IL-12-STAT4 signalling (Fig. 4E), seven of which showed robust expression (counts > 100), including triggering receptor expressed on myeloid cells 2 (TREM2), interferon regulatory factor 8 (IRF8), cytochrome b-245 alpha chain (CYBA), Coronin 1 A (CORO1A), transglutaminase 2 (TGM2), integrin subunit alpha M (ITGAM) and NCK-associated protein 1-like (NCKAP1L). RT-qPCR analysis revealed that the expression of TREM2 was significantly upregulated following IL-12 treatment (Fig. 4F, *p* < 0.05). In addition, the expression of other DEGs—including IRF8, CYBA, CORO1A, TGM2, ITGAM, and NCKAP1L—also increased significantly following IL-12 treatment (Fig. 4F; all *p* < 0.05). WB at the cellular level further confirmed increased TREM2 protein expression after IL-12 treatment (Figs. 4G, 4H, *p* < 0.05). Supplementary Fig. 6 presents the expression levels of TREM2 and DNAX-activating protein of 12 kDa (DAP12) under different IL-12 and glucose concentrations.

### IL-12 Increases TREM2 and DAP12 Levels in the Retinas of Diabetic Mice

**Fig. 5:**
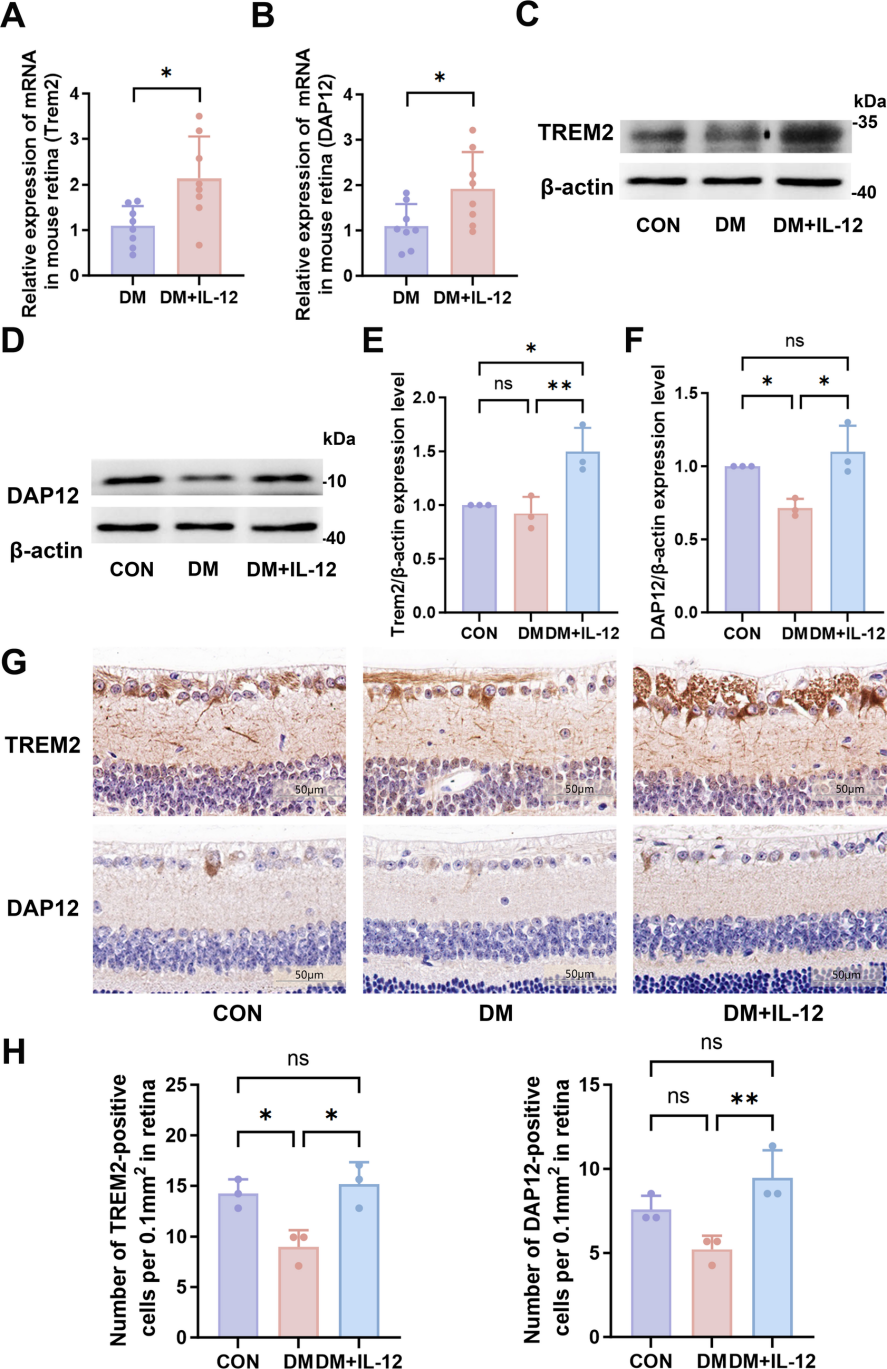
IL-12 increases TREM2 and DAP12 levels in the retinas of diabetic mice**.** (**A**, **B**) RT-qPCR analysis of TREM2 and DAP12 messenger RNA levels in the retinas of mice in the DM and DM + IL-12 groups; n = 8(TREM2: *p* = 0.0117, DAP12: *p* = 0.0277). (**C**–**F**) WB analysis of TREM2 and DAP12 protein levels in the retinas of mice in the CON, DM, and DM + IL-12 groups; n = 3(TREM2: CON vs. DM: *p* = 0.8182, CON vs. DM + IL-12: *p* = 0.0194, DM vs. DM + IL-12: *p* = 0.0099; DAP12: CON vs. DM: *p* = 0.0419, CON vs. DM + IL-12: *p* = 0.5409, DM vs. DM + IL-12: *p* = 0.0117). (**G**) Immunohistochemical analysis of TREM2 and DAP12 in the retinas of mice in the CON, DM, and DM + IL-12 groups; n = 3; scale bar = 50 μm. (**H**) Quantitative analysis of TREM2-positive and DAP12-positive cells in retina; n = 3 (TREM2: CON vs. DM: *p* = 0.0264, CON vs. DM + IL-12: *p* = 0.7968, DM vs. DM + IL-12: *p* = 0.0127; DAP12: CON vs. DM: *p* = 0.1021, CON vs. DM + IL-12: *p* = 0.1930, DM vs. DM + IL-12: *p* = 0.0098). Unpaired t-tests were performed to compare data between DM and DM + IL-12 groups. ANOVA tests were performed to compare data among CON, DM and DM + IL-12 groups. ns, not significant; *p < 0.05, **p < 0.01. CON, control group; DM, diabetic mice group; DM + IL-12, diabetic mice receiving intravitreal injection of IL-12; DAP12, DNAX-activating protein of 12 kDa; TREM2, triggering receptor expressed on myeloid cells. 2

RT-qPCR analysis demonstrated that intravitreal IL-12 injection significantly increased the mRNA levels of TREM2 (Fig. 5A, *p* < 0.05) and DAP12 (Fig. 5B, *p* < 0.05). WB results (Figs. 5C–5F) revealed significant differences in TREM2 and DAP12 protein levels among groups based on one-way ANOVA. Pairwise comparisons indicated higher TREM2 (Fig. 5E, *p* < 0.05) and DAP12 (Fig. 5F, *p* < 0.05) protein levels in the DM + IL-12 group compared with the DM group. Furthermore, immunohistochemical staining showed that both TREM2 and DAP12 were predominantly expressed in the retinal GCL, consistent with the typical localization of microglia (Fig. 5G). Additionally, IL-12 treatment induced a significant increase in the number of TREM2-positive and DAP12-positive cells in the retina (Fig. 5H, *p* < 0.05).

## Discussion

DR is a common and serious neurovascular complication of diabetes and a leading cause of vision loss worldwide [1, 2]. Although several treatments are available, most patients are diagnosed at the middle-to-late stages when retinal neural damage is often irreversible, limiting treatment effects[3, 30]. Therefore, identifying early therapeutic targets and strategies to halt DR progression and preserve visual function is critical. IL-12 has emerged as a potential therapeutic agent for early DRN[23]. This study examined the role and underlying mechanisms of IL-12 in DRN using a diabetic mouse model, with a focus on microglial phagocytic function.

Our findings demonstrated that STZ-induced diabetic mice developed early DRN, characterized by thinning of the NFL, GCL, and total retina, as early as 7 weeks after intraperitoneal STZ injection. Notably, these structural changes were partially reversed by intravitreal IL-12 injection. DRN precedes vascular lesions and represents a key early event in diabetic retinal pathology [28, 31]. Previous studies have shown that the NFL and GCL, which are primarily composed of RGCs, undergo significant thinning before vascular damage appears [32–34]. As the principal lesion cells in optic neuropathy, RGCs are vulnerable to damage, that can lead to vision loss. Because they lack self-renewal and have limited repair capacity, RGC injury is difficult to reverse, and often results in irreversible visual impairment [35]. Therefore, targeting the molecular mechanisms underlying RGC damage is clinically important for preserving visual function. Our results suggested that IL-12 mitigates early retinal neural injury in diabetes and protects RGCs from degeneration.

Notably, we found that the effect of IL-12 on DRN may be linked to microglial phagocytic function. RNA sequencing revealed that DEGs across the three groups were predominantly enriched in microglia, with KEGG analysis highlighting phagocytic pathways. Fluorescence staining confirmed microglial phagocytosis of dead RGCs, which was enhanced by IL-12 treatment. As innate immune cells in the retina, microglia rapidly and precisely clear dead cells, preventing inflammation caused by toxic intracellular components and maintaining retinal homeostasis [11–14]. IBA-1 immunohistochemistry and Cell Counting Kit-8 (CCK-8) assays showed that microglia were activated in the early stages of diabetes, and that IL-12 increased both their numbers and activation. These findings suggest that IL-12 enhances microglial phagocytic function, enabling efficient clearance of dead cells in the retina to prevent damage to the surrounding healthy RGCs, thereby preserving retinal function. A deeper understanding of microglial roles and interactions in the diabetic retina is essential for advancing treatment strategies. IL-12 shows strong potential as a targeted therapy to modulate microglial activity. However, elucidating the precise mechanisms underlying its effects on microglial behavior remains critical for clinical drugs development [12].

Our study suggests that IL-12 regulates phagocytosis through the JAK-STAT-TREM2 signaling pathway, which appears to be the primary mechanism underlying IL-12-mediated activation of microglial phagocytic function. The cellular effects of IL-12 are mediated via the classical STAT4 phosphorylation pathway. Upon binding to the IL-12 receptor on the cell membrane, IL-12 activates tyrosine kinases JAK2 and TYK2, which in turn phosphorylate and activate STAT4 [36–38]. WB confirmed that IL-12 promotes STAT4 phosphorylation in microglia. Notably, further bioinformatics analysis and RT-qPCR experiments revealed that IL-12-induced STAT4 activation upregulated phagocytosis-related genes, including TREM2. Previous studies have shown that the TREM2/DAP12 pathway mediates non-inflammatory phagocytosis by microglia. TREM2 signaling promotes the phosphorylation of DAP12, which regulates membrane dynamics and cytoskeletal reorganization, facilitating the phagocytosis of apoptotic cells and axonal debris from damaged neurons and promoting axonal regeneration [39–41]. Both in vitro and in vivo experiments demonstrated that IL-12 upregulates the TREM2/DAP12 pathway on microglial membranes, enhancing phagocytic and proliferative activity. We therefore speculate that, in the high-glucose environment of early DR, RGCs undergo apoptosis, and IL-12 activates microglial phagocytic function via the JAK-STAT-TREM2 pathway to clear dead RGCs, thereby inhibiting the progression of DRN and alleviating early DR.

This study has several limitations. We recognize that our study is not sufficiently innovative and requires further exploration in future research. First, although IL-12 upregulates the TREM2/DAP12 pathway in microglia suppressed by high glucose levels, the relationship between TREM2 downregulation and impaired phagocytic function remains unclear. Second, IL-12 enhances microglial phagocytic function and reduces RGC damage in a high-glucose environment, supporting its potential as a therapeutic agent in clinical settings, however, its effects on overall retinal homeostasis under such conditions remain to be clarified. Third, the absence of direct evidence for neuronal injury in vivo—both structural (e.g., spectral-domain optical coherence tomography, SD-OCT) and functional (e.g., electroretinography, ERG) limits direct translation to therapeutic potential—an issue we are addressing in ongoing study. Fourth, in-vivo phagocytosis assays are still needed to connect IL-12-enhanced microglial phagocytosis to neuroprotection. Moreover, further research is required to develop long-term, sustainable treatments for early DR that maintain efficacy without compromising patient comfort. Additionally, although our controlled experiments support IL-12’s neuroprotective role, we cannot entirely exclude the potential influence of injection-related effects, prompting further optimization of delivery methods.

In conclusion, this study demonstrates that IL-12 upregulates the TREM2/DAP12 pathway on the microglial membrane, enhancing phagocytic function and mitigating early neural injury in DR. These findings offer new therapeutic targets and strategies to inhibit early DR progression.

## Supplementary Information

Below is the link to the electronic supplementary material.

Supplementary file1 (DOCX 1538 KB)

## Data Availability

The primary data generated or analyzed during this study are included in the main manuscript and additional files. Raw data have been deposited to National Center for Biotechnology Information (NCBI) under the BioProject number PRJNA1266371.
