# Supplementary material for: IL-12 ameliorates diabetic retinal neurodegeneration by activating microglial phagocytosis via the TREM2/DAP12 pathway
Source: Mol Neurobiol. 2025 Nov 29;63(1):215. doi: 10.1007/s12035-025-05512-1 (PMC12664847; doi:10.1007/s12035-025-05512-1)
Supplement: Supplementary file 1 — Supplementary file1 (DOCX 1538 KB) [file 12035_2025_5512_MOESM1_ESM.docx]

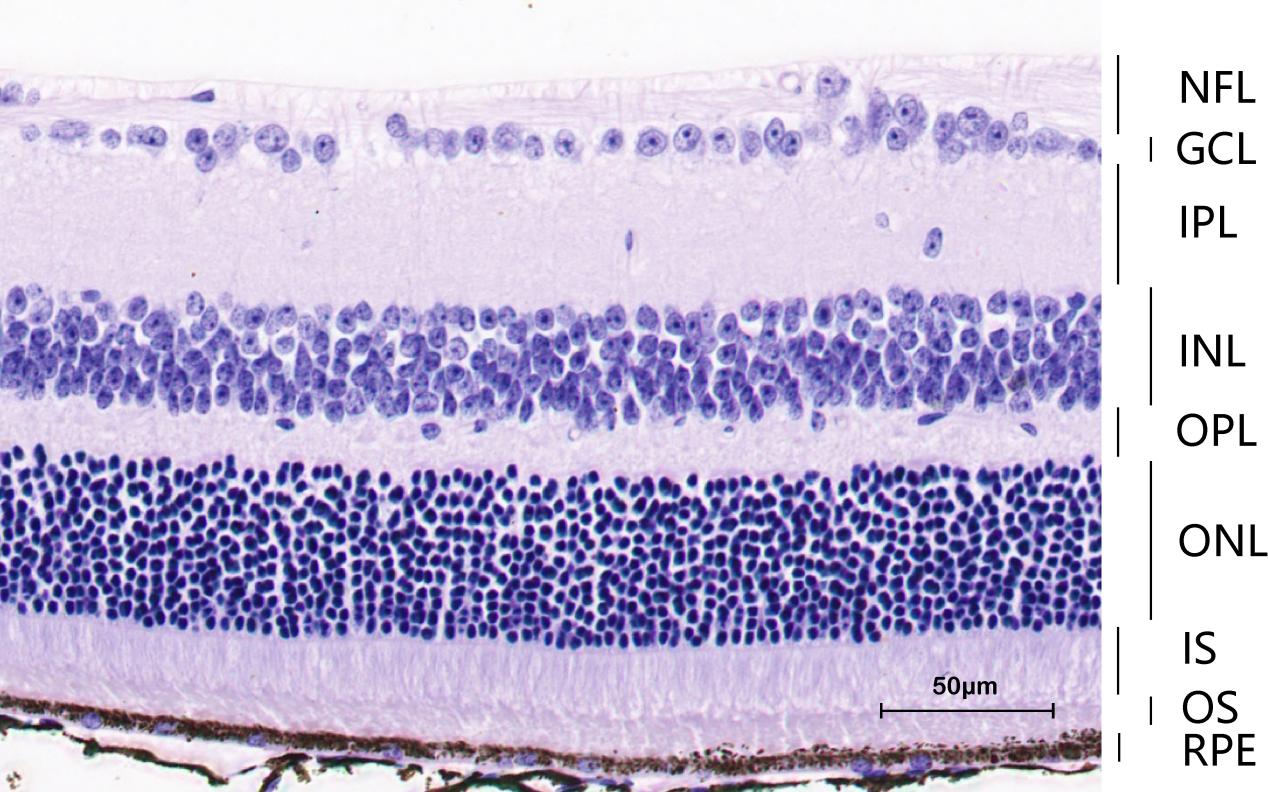


**Supplementary Fig. 1. Schematic diagram of mouse retinal layers.**

HE staining of mouse retina paraffin section, ×400. NFL is the nerve fiber layer, GCL is the ganglion cell layer, IPL is the inner plexiform layer, INL is the inner nuclear layer, OPL is the outer plexiform layer, ONL is the outer nuclear layer, IS is the inner segment of photoreceptors, OS is the outer segment of photoreceptors, RPE is the retinal pigment epithelium layer.


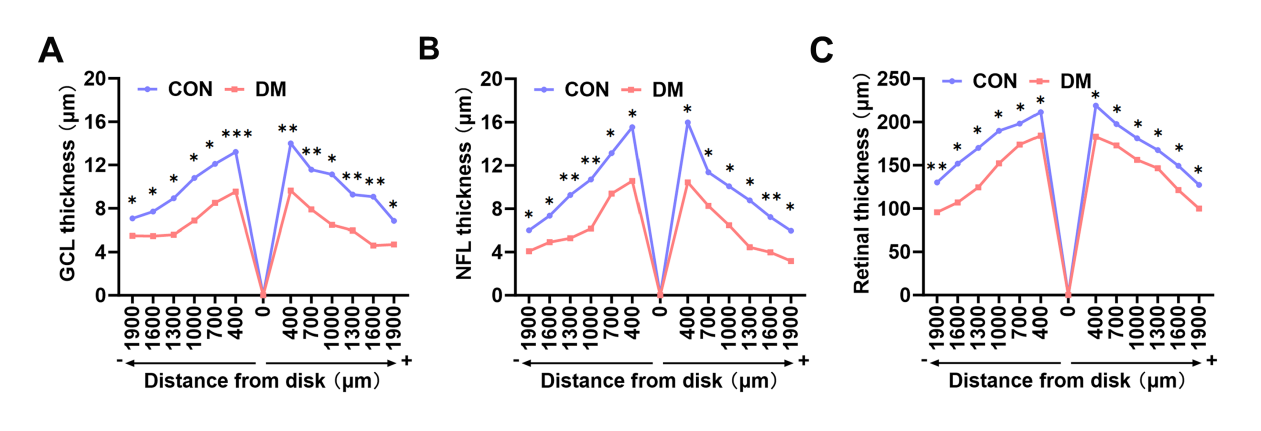


**Supplementary Fig. 2. Diabetic mice shows retinal neurodegeneration.**

1. C)Measurement and analysis of GCL, NFL and the entire retinal thickness at different distances from the optic nerve root in CON and DM group. n=3. **p*<0.05, ***p*<0.01, ****p*<0.001.


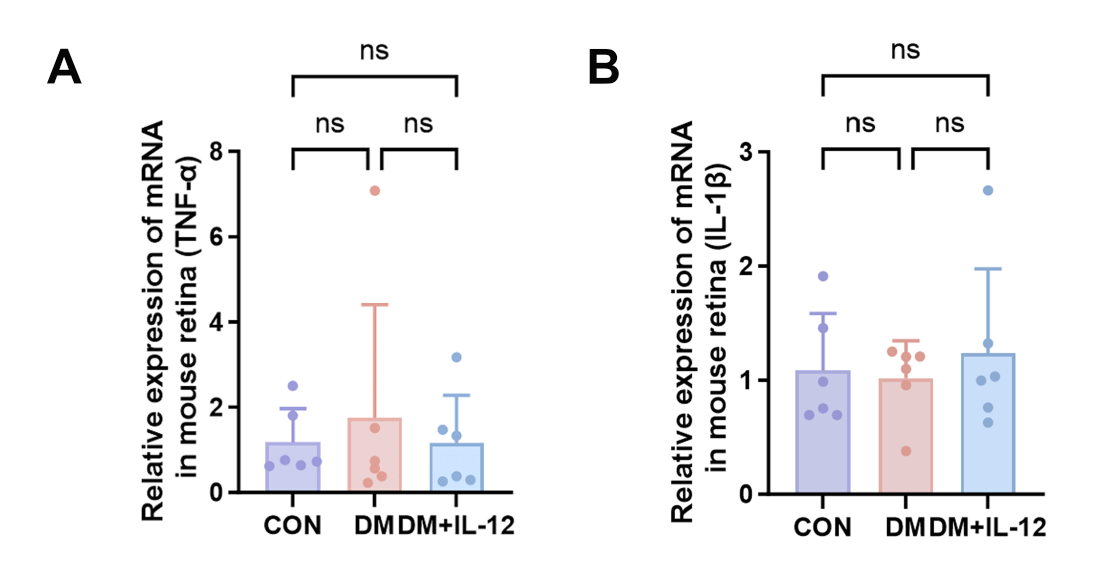


**Supplementary Fig. 3. Early-stage diabetic retinopathy shows no overt inflammation, and IL-12 does not elicit inflammation.**

(A, B) RT-qPCR analysis of TNF-α and IL-1β in the retinas of mice in the CON, DM and DM + IL-12 groups; n = 6; ns, not significant.

**
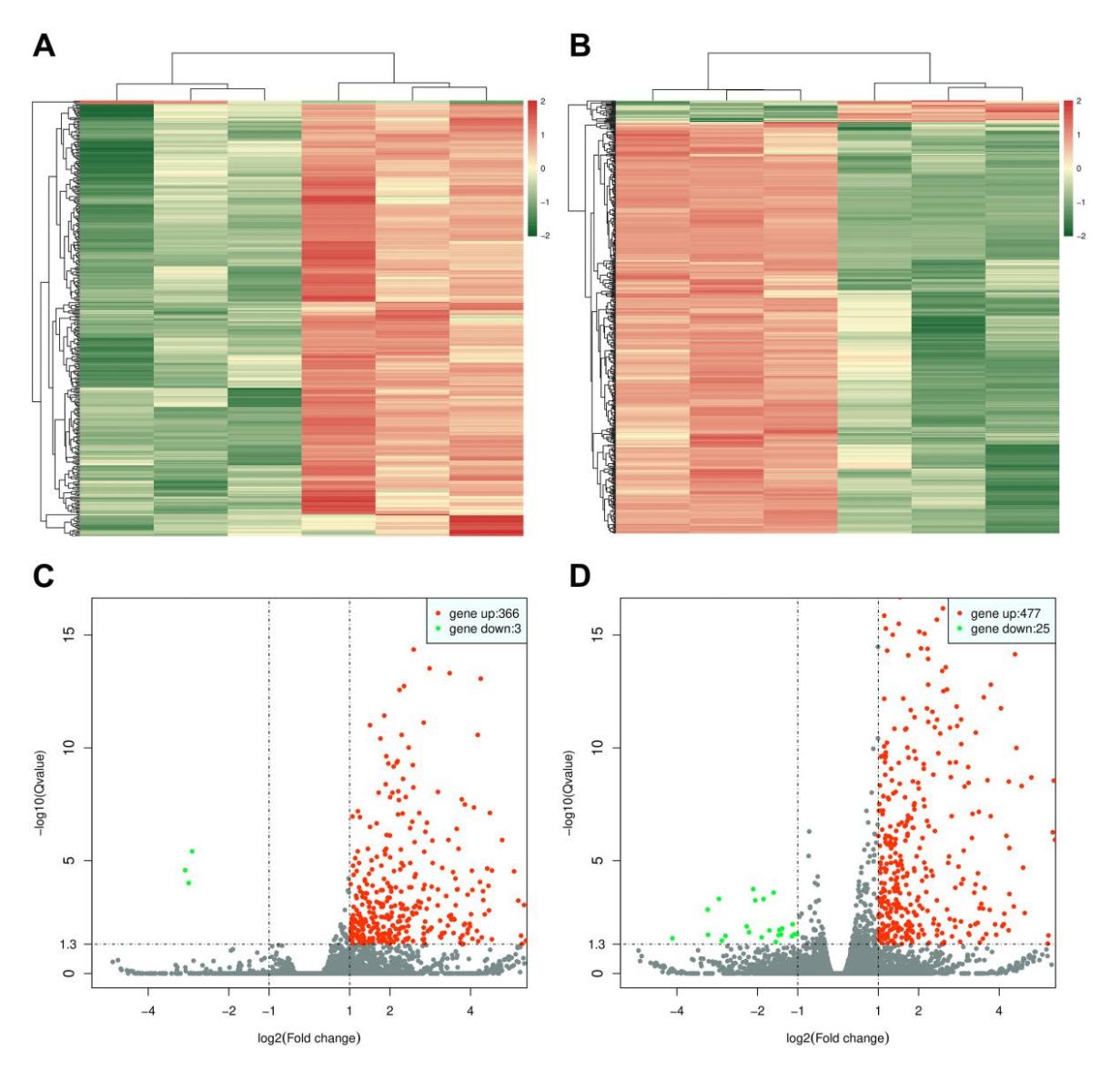
**

**Supplementary Fig. 4. Heat maps and volcanic maps of RNA sequencing.**

(A) and (B) are the heat maps showing DEGs in the retina of mice in DM group versus CON group,DM + IL-12 group versus DM group, and the color represents the log10 (expression+1) , the red rectangles represent significantly up-regulated genes, and green represents significantly down-regulated genes. n=3. (C) and (D) are the volcanic maps showing DEGs in the retina of mice in DM group versus CON group, DM + IL-12 group versus DM group, and the abscissa represents the change of gene expression multiple in different groups, while the ordinate represents the statistical significance of the difference; red dots represent significantly up-regulated genes, and green represents significantly down-regulated genes, n=3.

DEGs, differentially expressed genes; CON, control group; DM, diabetic mice group; DM + IL-12, diabetic mice receiving intravitreal injection of IL-12.


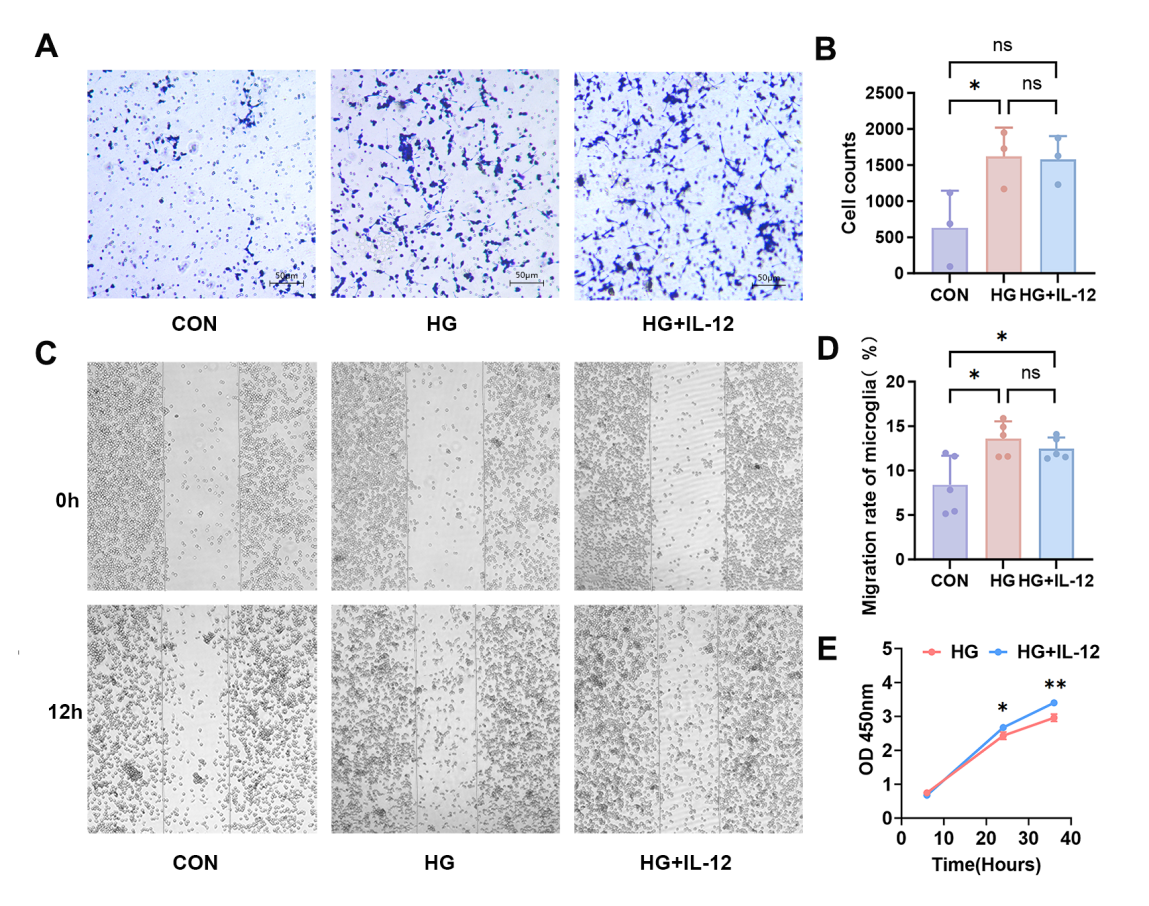


**Supplementary Fig. 5. IL-12 enhances the proliferative capabilities of microglias in a high-glucose environment, and shows no impact on their migration.**

(A-B) Migration of microglia in CON, HG and HG+IL-12 group in Transwell experiment, n=3. ns, not significant, **p*<0.05. (C-D) Migration of microglia in CON, HG and HG+IL-12 group 0 and 12 hours after the intervention in cell scratch test, n=5. ns, not significant, **p*<0.05. (E)The proliferative ability of microglia in HG and HG+IL-12 group detected by CCK8 assay, n=3.

ns, not significant, **p*<0.05, ***p*<0.01.


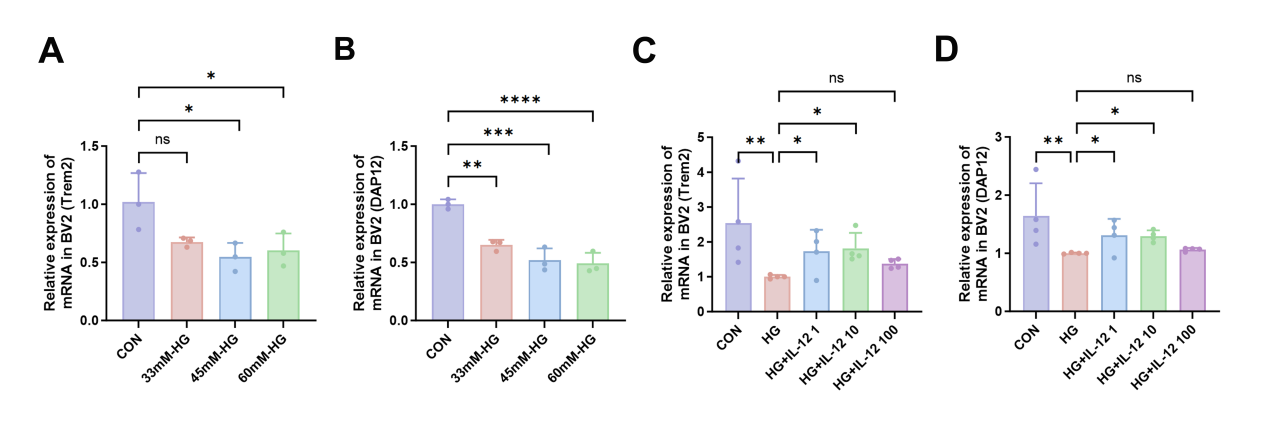


**Supplementary Fig. 6. IL-12 increases TREM2 and DAP12 levels in microglia suppressed by a high-glucose environment.**

1. B)RT-qPCR analysis of TREM2 and DAP12 of in microglia in the CON group and HG group with different glucose concentration, n=3. (C-D)RT-qPCR analysis of TREM2 and DAP12 of in microglia in the CON group, HG group and HG group with different IL-12 concentration, n=3.

ns, not significant, **p*<0.05, ***p*<0.01, ****p*<0.001, *****p*<0.0001.
